# Integration of liquid biopsy and immunotherapy: opening a new era in colorectal cancer treatment

**DOI:** 10.3389/fimmu.2023.1292861

**Published:** 2023-11-21

**Authors:** Shiya Yao, Yuejun Han, Mengxiang Yang, Ketao Jin, Huanrong Lan

**Affiliations:** ^1^ Department of Colorectal Surgery, Affiliated Jinhua Hospital, Zhejiang University School of Medicine, Jinhua, Zhejiang, China; ^2^ Department of Surgical Oncology, Hangzhou Cancer Hospital, Hangzhou, Zhejiang, China

**Keywords:** liquid biopsy, immunotherapy, circulating tumor DNA (ctDNA), Circulating tumor cells (CTC), colorectal cancer, neoantigen, mass spectrometry, flow cytometry

## Abstract

Immunotherapy has revolutionized the conventional treatment approaches for colorectal cancer (CRC), offering new therapeutic prospects for patients. Liquid biopsy has shown significant potential in early screening, diagnosis, and postoperative monitoring by analyzing circulating tumor cells (CTC) and circulating tumor DNA (ctDNA). In the era of immunotherapy, liquid biopsy provides additional possibilities for guiding immune-based treatments. Emerging technologies such as mass spectrometry-based detection of neoantigens and flow cytometry-based T cell sorting offer new tools for liquid biopsy, aiming to optimize immune therapy strategies. The integration of liquid biopsy with immunotherapy holds promise for improving treatment outcomes in colorectal cancer patients, enabling breakthroughs in early diagnosis and treatment, and providing patients with more personalized, precise, and effective treatment strategies.

## 1 Introduction

Colorectal cancer (CRC) ranks as the second leading cause of cancer-related mortality worldwide (1), with increasing incidence and mortality rates. Most patients with metastatic CRC receive systemic drug therapy, which can prolong survival and improve symptoms but generally falls short of achieving a cure, making long-term survival challenging (2). In recent years, immunotherapy, represented by immune checkpoint inhibitors (ICIs), has revolutionized the traditional treatment approaches for CRC (3–5).

Immunotherapy has emerged as a promising approach for treating various cancers, including CRC. However, a challenge in the field of immunotherapy is the accurate assessment of treatment response and monitoring the effectiveness of immune interventions. Some biomarkers have been identified as predictors of the anti-tumor efficacy of ICIs, but there remains a need for clinically useful biomarkers. Traditional response assessment criteria, such as tissue biopsies, fail to capture the complex dynamics of the immune system and tumor microenvironment(TME) (6, 7), highlighting the urgent need for novel detection methods to monitor the efficacy of immunotherapy in real time and enable timely treatment adjustments (8–10).

With the rapid advancements in cell isolation and genetic testing technologies, liquid biopsy, which involves minimally invasive acquisition of tumor material, has gained recognition for its importance in precision oncology (11–13). It allows real-time monitoring of tumor progression, recurrence, or treatment response at the molecular level (14, 15). Circulating tumor cells (CTCs) and circulating tumor DNA (ctDNA) have emerged as representative liquid biopsy biomarkers (16).

In this review, we will first discuss the current biomarkers used for immune monitoring in CRC. Secondly, we will analyze the recent research progress in liquid biopsy, specifically focusing on ctDNA and CTCs, as adjuncts for CRC treatment. Finally, we will discuss the potential of novel technologies to address the challenges of immune therapy monitoring by providing solutions for liquid biopsy in the context of adjuvant immunotherapy.

## 2 Biomarkers currently used for immunotherapy monitoring in CRC

Currently, the treatment modalities for CRC include endoscopic and surgical resection, systemic adjuvant chemotherapy, radiotherapy, targeted therapy, and immunotherapy (1, 17). Over the past five years, the discovery of ICIs and the successful use of ICIs have revolutionized the treatment paradigm for CRC. ICIs have brought new opportunities for the treatment of CRC (18–21). In 2017, the U.S. Food and Drug Administration (FDA) approved the use of immune therapy drugs for the treatment of metastatic colorectal cancer (mCRC) (22–25). Pembrolizumab, an anti- programmed death receptor 1(PD-1) monoclonal antibody, has been established as the first-line treatment standard for microsatellite-high/deficient mismatch repair (MSI-H/dMMR) mCRC (5). Immunotherapy is gradually becoming an essential component of precision treatment for mCRC.

With the continuous development of medical science and technological advancements, biomarkers play an increasingly important role in clinical applications. These biomarkers provide crucial information to assist physicians in the diagnosis, treatment, and monitoring of diseases. Some biomarkers, such as programmed cell death ligand 1 (PD-L1), tumor mutational burden (TMB), and microsatellite stability, have been identified as predictors of the anti-tumor efficacy of ICIs. However, there remains a gap in the clinical demand for effective biomarkers (26).

Microsatellite stability is currently the most relevant biomarker for immunotherapy sensitivity in CRC and is typically evaluated through solid tissue specimens (27, 28). MSI is a condition of genetic instability caused by defects in DNA repair mechanisms and is commonly observed in a subset of CRC patients. However, despite the promising prospects of MSI-H/dMMR as a biomarker for immunotherapy in CRC, there is variability in the reported overall response rates (ORR) in MSI-H mCRC patients, ranging from 30% to 70% (29–32). This suggests that a significant number of MSI-H mCRC patients do not benefit from immunotherapy (33). Conversely, a small subset of microsatellite-stable (MSS) CRC patients exhibit a response to immunotherapy. One contributing factor to this phenomenon is diagnostic errors caused by the detection methods (34).

Currently available methods for detecting microsatellite instability include immunohistochemistry (IHC), polymerase chain reaction (PCR), and next-generation sequencing (NGS). Immunohistochemistry detects the expression of four mismatch repair genes (MLH1, MSH2, MSH6, and PMS2) in the nuclei of tumor cells, and the absence of one or more of these proteins is defined as dMMR, otherwise known as proficient mismatch repair (pMMR) (35). Detection of MSI status is accomplished through immunohistochemistry on tissue specimens, which has the limitations of subjectivity and a lack of uniform standards (36).

TMB is associated with the treatment response to immunotherapy, and elevated plasma TMB levels (≥28 Mut/Mb) have shown predictable responses to the combination therapy of PD-L1 inhibitor durvalumab and CTLA4 inhibitor tremelimumab in MSS CRC patients (37). TMB has been approved by the U.S. FDA as a diagnostic biomarker for the use of pembrolizumab or dostarlimab in cancer immunotherapy (38, 39). Furthermore, studies have shown that high TMB (TMB ≥8 Mut/Mb) in CRC patients is associated with longer overall survival (OS) and better prognosis compared to low TMB (34, 40). However, the use of TMB as a sole predictor of immunotherapy response in CRC remains controversial. Limitations of using TMB as a predictive biomarker for immunotherapy response in CRC were observed in the KEYNOTE 177 trial (41). TMB assessment requires tumor tissue specimens as the gold standard, and tumor heterogeneity poses limitations to its precise estimation (42). Additionally, similar to any other gene or genomic biomarker, TMB may undergo changes in CRC following standard cytotoxic drug treatments (43).

**Figure 1:**
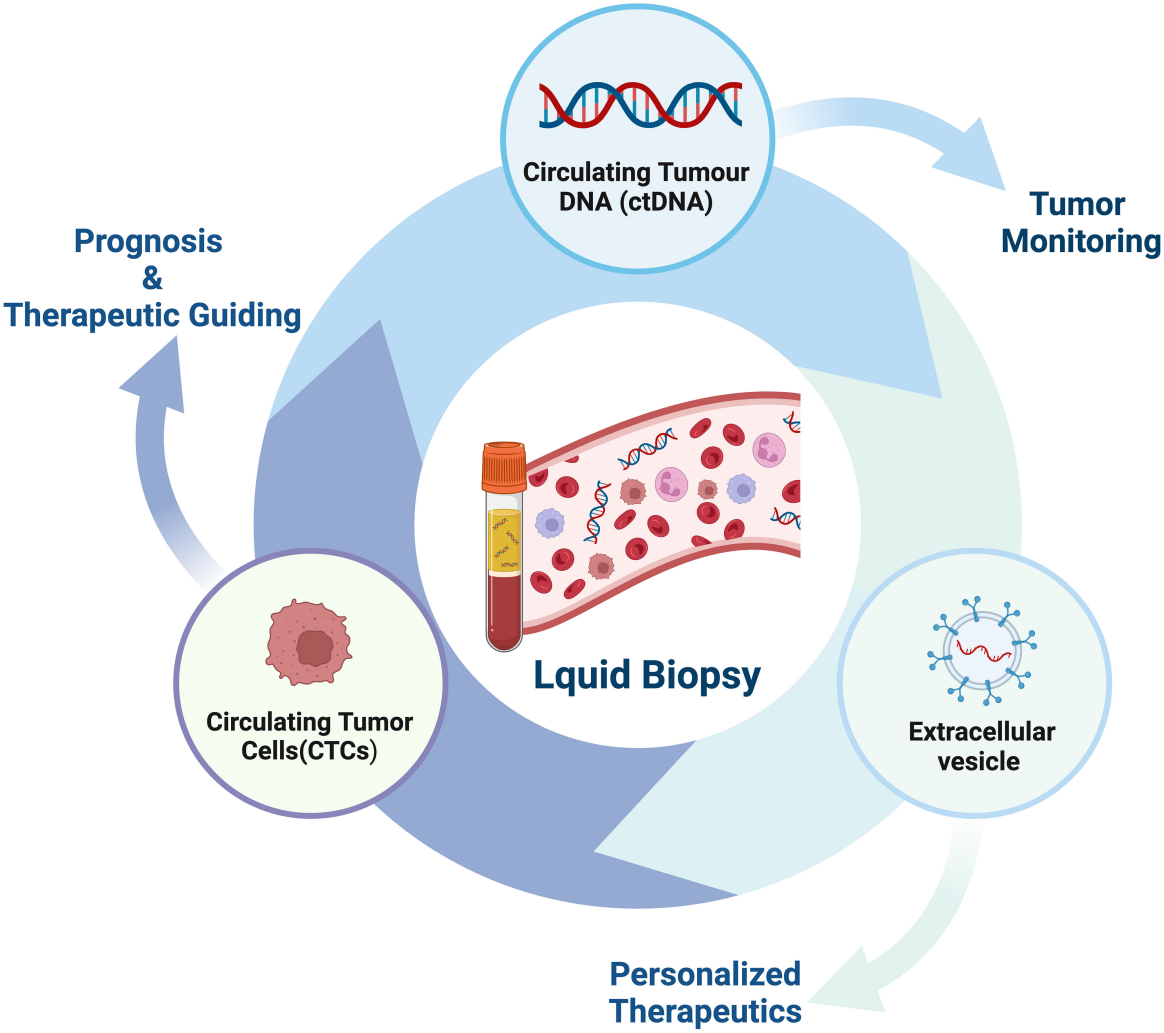
Programmed cell death protein 1 (PD-1) plays a crucial role in the initiation and effector phases of the anti-tumor immune response. T cell activation is a fundamental process in immune response, involving antigen presentation by dendritic cells and recognition by the T cell receptor (TCR). Once activated, T cells migrate to the tumor site to eliminate malignant cells. However, the tumor or bystander cells such as macrophages may upregulate PD-L1, which hinders T cell function by inducing inhibitory intracellular signaling.

Moreover, PD-L1 expression levels serve as important indicators of the immune status in cancer patients, which reflects the tumor’s response to immunotherapy (44–46). In certain solid tumors such as non-small cell lung cancer, melanoma, and renal cell carcinoma, PD-L1 expression has been proposed as a predictive biomarker for immunotherapy response (47–49). High PD-L1 expression is associated with a better response to immunotherapy. Tumor cells induce immune evasion by upregulating the expression of PD-L1, which binds to PD-1 on the surface of T cells, leading to T cell inactivation (**Figure 1**). ICIs block the interaction between PD-1 and PD-L1, thereby reactivating the body’s anti-tumor immune response (47). CRC patients have been reported to exhibit positive PD-L1 expression (50, 51). Although high PD-L1 expression is associated with a favorable prognosis in CRC patients (52–54), current clinical data suggest that the use of PD-L1 expression alone cannot accurately predict the immunotherapy response in CRC (55).

However, despite the widespread application of certain biomarkers, we still face various challenges and limitations. To overcome these limitations, researchers are actively searching for more suitable detection methods and therapies to enhance the reliability and effectiveness of biomarkers in clinical practice. Through continuous exploration and innovation, we hope to open up new fields and approaches that will bring greater breakthroughs in disease prevention, diagnosis, and treatment. Therefore, the quest for more accurate and reliable biomarkers has become a hot topic in medical research, offering new opportunities and hopes for improving patient health outcomes.

## 3 Application of liquid biopsy in adjuvant therapy for CRC

**Figure 2:**
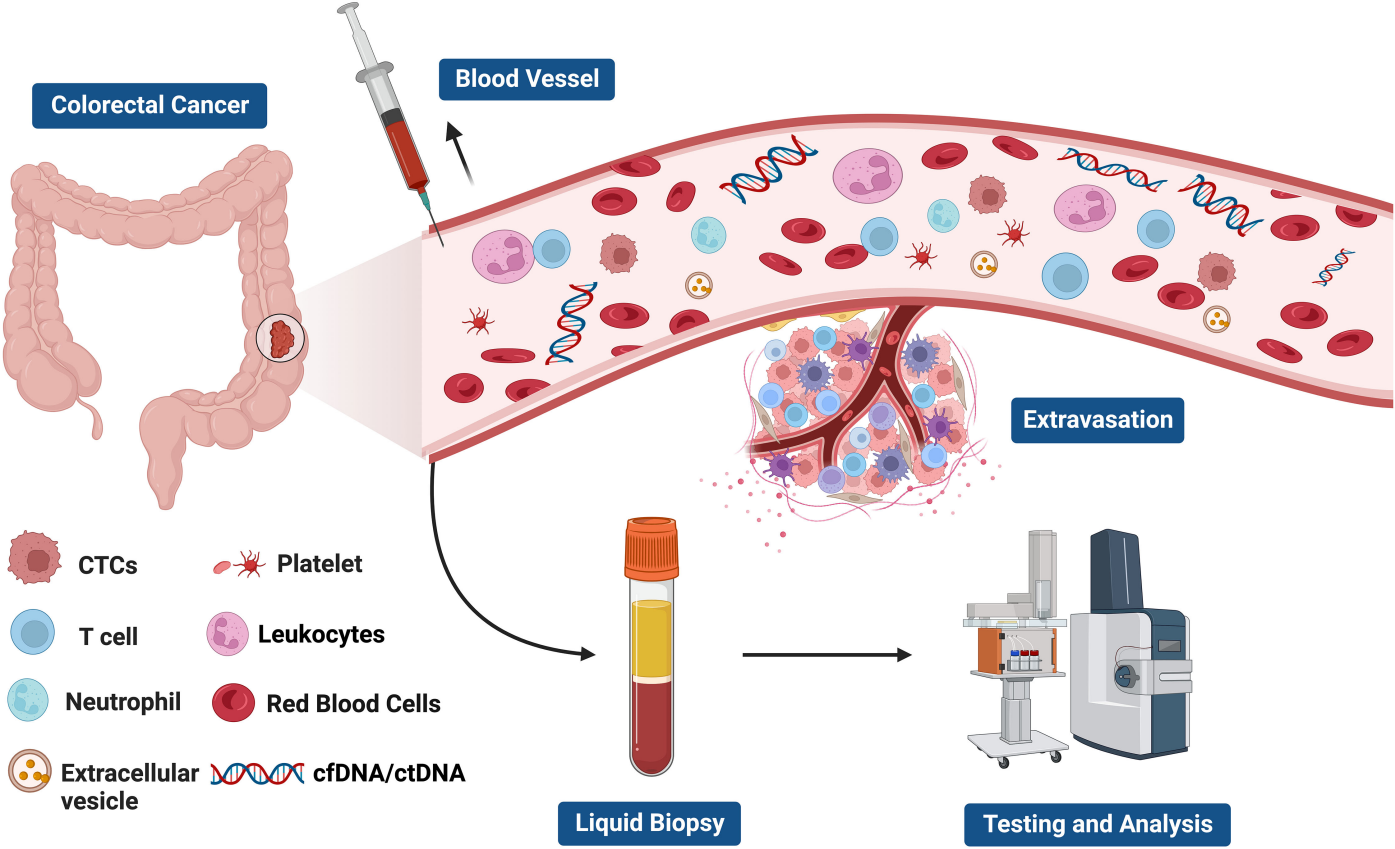
The current clinical applications of liquid biopsy. Liquid biopsy provides a non-invasive approach to assess the dynamic changes and treatment response of tumors by analyzing components such as circulating tumor DNA (ctDNA), circulating tumor cells (CTCs), and extracellular vesicles. These components can be obtained from blood samples and detected using highly sensitive analytical techniques. The information derived from liquid biopsy aids in guiding personalized treatment strategies, including the selection of appropriate drugs, monitoring treatment efficacy, and providing treatment guidance. Liquid biopsy holds promising applications in tumor management, offering patients more accurate and effective treatment choices.

**Figure 3:**
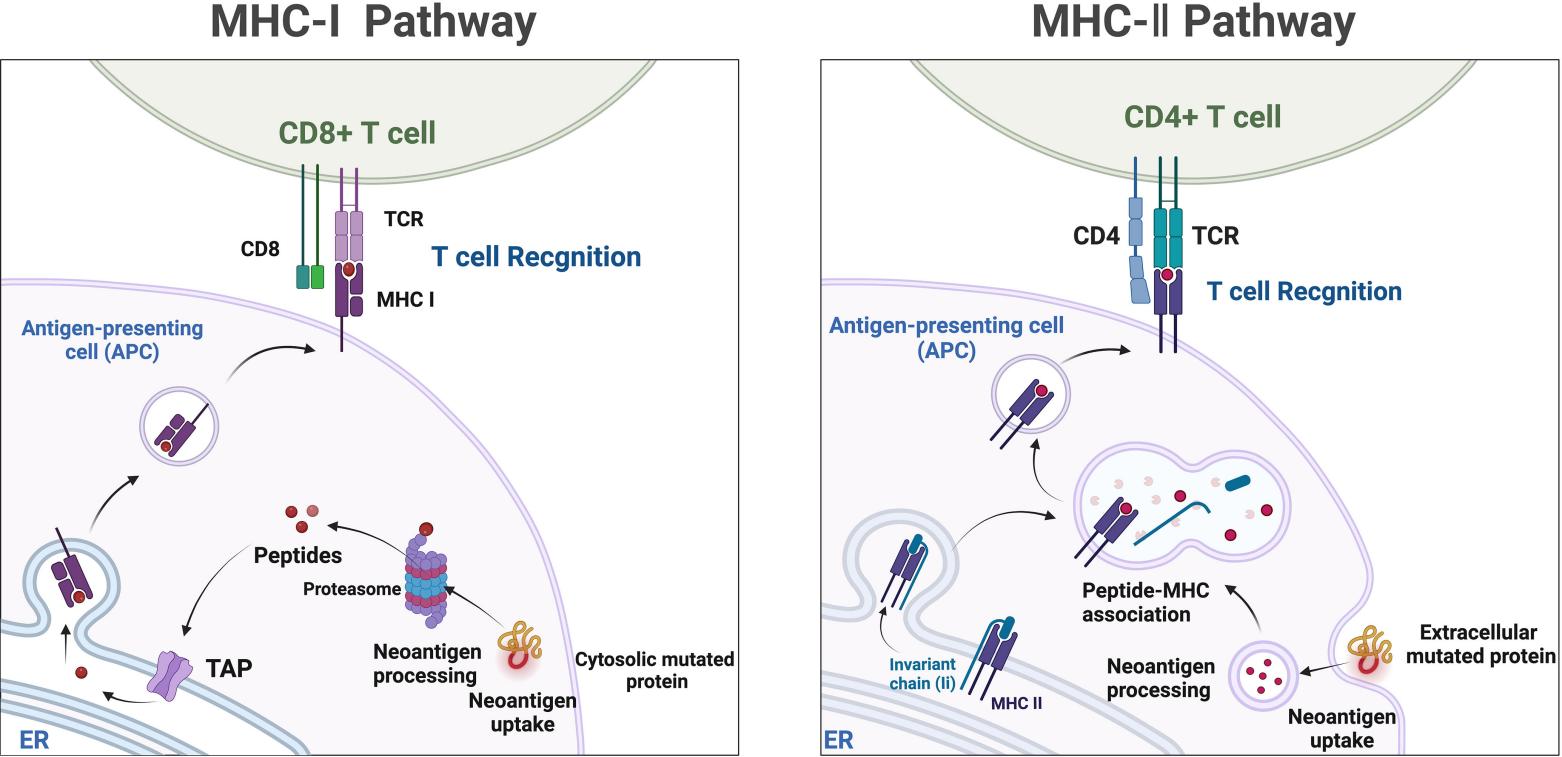
The components of liquid biopsy in colorectal cancer(CRC). circulating tumor cells (CTCs) and Circulating tumor DNA (ctDNA) are the main constituents of current liquid biopsy approaches. ctDNA, extracellular vesicle, and CTCs are shed directly from tumor masses or metastatic lesions into the bloodstream. After collection of blood samples, further analysis of these components provides a comprehensive tumor characterization.

Liquid biopsy has opened up a new avenue for cancer patients in terms of prognostic evaluation, detection of minimal residual disease (MRD), treatment selection, resistance mechanisms and monitoring, as well as early cancer diagnosis (56–61) (**Figure 2**). The fundamental principle of liquid biopsy is the non-invasive detection and assessment of tumors using circulating cell-free DNA (cfDNA), RNA, or tumor cells present in bodily fluids such as blood, urine, and cerebrospinal fluid (62–66). CTCs and ctDNA are important components and are generally considered the foundation of liquid biopsy. ctDNA is formed by apoptotic and necrotic tumor cells, which release fragmented DNA into the bloodstream and harbor genetic alterations of the original tumor cells (67, 68). CTCs are cancer cells that spontaneously detach from primary or metastatic tumors and circulate in the bloodstream (69). They serve as “seeds” of the tumor and can contribute to recurrence through liver metastasis, lymphatic dissemination, and angiogenesis (**Figure 3**).

Certain characteristics of CTCs, such as the expression of surface markers or genetic mutations, are associated with the prognosis of cancer patients. Changes in CTC counts are correlated with shortened disease-free survival (DFS), progression-free survival (PFS) and OS (70, 71). Increased levels of ctDNA may indicate disease progression (72, 73). By regularly monitoring changes in CTCs and ctDNA, the effectiveness of treatment and the dynamic changes of the tumor can be assessed (59).

MRD refers to the presence of extremely low levels of cancer cells or cancer-associated genetic material after completion of treatment (74). Early detection of MRD can be achieved through the detection of CTCs and ctDNA (75–77). According to the latest results from the GALAXY observational study presented at the 2023 ASCO conference, the detection of MRD through ctDNA testing at 4 weeks post-surgery is the strongest prognostic risk factor for DFS in stage II to IV CRC patients, regardless of BRAF ^V600E^ or MSI status (78, 79).

In early-stage CRC patients, the presence of ctDNA positivity after curative surgery is associated with a higher risk of disease recurrence (74, 80–85). A study demonstrated that ctDNA positivity after adjuvant chemotherapy is associated with poorer DFS, and ctDNA detection precedes radiological relapse by a median of 11.5 months (86). The DYNAMIC trial (87) investigated whether a ctDNA-guided approach could reduce the use of adjuvant therapy without compromising the risk of recurrence compared to the standard approach in stage II CRC. Among the 455 randomly assigned patients, 302 were assigned to the ctDNA-guided management group, and 153 were assigned to the standard management group. The median follow-up time was 37 months. The proportion of patients receiving adjuvant chemotherapy was lower in the ctDNA-guided group compared to the standard management group (15% vs. 28%). The ctDNA-guided management was non-inferior to standard management in terms of 2-year disease-free survival rates (93.5% vs. 92.4%). The 3-year disease-free survival rate was 86.4% in ctDNA-positive patients receiving adjuvant chemotherapy and 92.5% in ctDNA-negative patients not receiving adjuvant chemotherapy. The ctDNA-guided approach can reduce the use of adjuvant chemotherapy without compromising disease-free survival in the treatment of stage II CRC. In various cohorts of non-mCRC and resected colorectal liver metastasis patients, the proportion of disease recurrence has consistently exceeded 80% in patients with detectable ctDNA who did not receive adjuvant therapy (74, 88, 89). A study (90)demonstrated that preoperative ctDNA could be detected in 108 out of 122 (88.5%) patients with stage I to III CRC. Longitudinal ctDNA analysis identified 14 out of 16 (87.5%) recurrences after definitive treatment. Furthermore, at postoperative day 30, ctDNA-positive patients were more likely to experience recurrence compared to ctDNA-negative patients. Another study (91) evaluated the prognostic impact of postoperative ctDNA in stage I-III CRC patients and found that ctDNA status was the most significant and independent factor in predicting recurrence-free survival (RFS). Postoperative plasma samples from 108 patients underwent NGS quality control, with 17 (15.7%) classified as ctDNA-positive and 91 classified as ctDNA-negative. Among these 17 ctDNA-positive patients, 2 were stage II, and 15 were stage III. The recurrence rate for ctDNA-positive patients was 76.5% (13/17), significantly higher than the 16.5% (15/91) in ctDNA-negative patients. Kaplan-Meier survival curves showed significantly poorer recurrence-free survival (RFS) for ctDNA-positive patients compared to ctDNA-negative patients. The study results also demonstrated a sensitivity of 49.6% and specificity of 94.7% for ctDNA alone in predicting 2-year RFS. A predictive model combining ctDNA with clinical-pathological risk factors, referred to as CTCP prediction model, exhibited better RFS predictive value than ctDNA alone in stage I-III CRC patients and increased the sensitivity for 2-year RFS to 87.5%. The predictive value of this model was also externally validated. Additionally, ctDNA can be utilized for monitoring locally advanced rectal cancer (LARC) patients who achieve complete response after neoadjuvant therapy and adopt an “watch-and-wait” strategy (92, 93).

Precision therapy involves customizing drug treatments based on the individual characteristics of tumors (94). Liquid biopsy provides molecular profiling information of tumors, such as gene mutations and chromosomal rearrangements, to select appropriate targeted therapy drugs. In CRC, analysis of the molecular features of individual CTCs has revealed significant heterogeneity in the presence of EGFR mutations and other genetic mutations associated with EGFR inhibition (such as KRAS and PIK3CA mutations) among patients and between patients, which explains the different response rates to EGFR-targeted therapy (95). By analyzing mutations in ctDNA, patients who may benefit from targeted EGFR therapy or BRAF and MEK inhibitors can be identified (96–98). In ctDNA-positive CRC patients, plasma testing for RAS status demonstrated a sensitivity of 92.9% and specificity of 87.7% (99). The CHRONOS trial confirmed the importance of evaluating RAS status using ctDNA in metastatic CRC patients (100, 101). The study found that patients who were mutation-negative in ctDNA had good clinical responses to anti-EGFR retreatment. An ongoing randomized Phase III trial (102) is expected to reveal that liquid biopsy-based retreatment with anti-EGFR monoclonal antibodies achieves approximately one-third objective responses in mCRC patients, prospectively demonstrating the effective management of patients through genetic profiling using liquid biopsy.

Similar to predicting response to chemotherapy and/or targeted therapy, liquid biopsy based on ctDNA can guide immunotherapy. While immune therapy has prolonged PFS in patients with MSI-H CRC, it is interesting to note that in the KEYNOTE-177 study, approximately 30% of patients showed no response to pembrolizumab (32). Using ctDNA monitoring to identify non-responders at an early stage can provide an opportunity for physicians to switch to chemotherapy or consider the addition of anti-CTLA-4 agents (103). The mutational burden in ctDNA is associated with the efficacy of immunotherapy and serves as a direct reflection of tumor burden (104–110). Liquid biopsy utilizes ctDNA released into the bloodstream, providing a non-invasive alternative. However, similar to TMB, the MSI status is also influenced by spatial and temporal heterogeneity, making it challenging to monitor its therapeutic value through liquid biopsy (111). A study used liquid biopsy to detect the MSI status in ctDNA and found a high concordance with results from traditional tissue biopsy, effectively predicting immunotherapy sensitivity and clinical outcomes in patients (112). Another study demonstrated that liquid biopsy could monitor changes in MSI status at different time points, providing important information on treatment response and disease progression in patients (74). Furthermore, recent studies have proposed that the concentration of cfDNA can serve as a predictive biomarker for immune therapy response (113–116). cfDNA can be detected in MSI-H CRC patients who respond well to immunotherapy (117, 118). Moreover, dynamic changes in ctDNA have been shown to predict the efficacy of other immunotherapies, including chimeric antigen receptor T-cell (CAR-T) therapy (119, 120). Analysis of tumor-derived structural alterations through shallow whole-genome sequencing revealed a decrease in ctDNA levels in patients who responded well to CAR-T cell therapy, while an increase was observed in patients who did not achieve a treatment response. The abundance of CAR-T cell construct-derived DNA in peripheral blood may be correlated with the dynamic changes in ctDNA and can be used in combination (121).

Several clinical trials focusing on liquid biopsy in the context of immunotherapy are currently underway. The ongoing ARETHUSA trial (NCT03519412) is investigating the use of ctDNA-based TMB assessment as a predictive marker for immunotherapy response following pretreatment with temozolomide in MGMT-methylated mCRC (122). It is worth noting that there are ongoing efforts to identify the optimal approach for TMB analysis (123). The use of ctDNA for predicting response to immunotherapy has shown promise in the INSPIRE study, a prospective Phase II trial that conducted serial ctDNA assessments in 94 patients with advanced solid tumors receiving pembrolizumab (124). It was found that in 42% of patients, an increase in ctDNA and tumor volume was observed at 6 weeks, accurately predicting lack of response with 100% specificity. During immunotherapy, 16% of patients exhibited ctDNA clearance, with a median follow-up exceeding 25 months and an OS of 100%. At the start of the third treatment cycle, 98% of patients had an increase in ctDNA, indicating lack of objective response. This may enable the avoidance of ineffective treatment in a subset of patients. Zhang et al. characterized the prognostic and predictive impact of ctDNA in patients with 16 different solid tumor types enrolled in Phase I/II trials of single-agent durvalumab or combination therapy with tremelimumab (125). Higher pretreatment variant allele frequency (VAF) was associated with poorer survival but not with ORR. In contrast, reductions in VAF during treatment were associated with prolonged PFS, OS, and ORR, suggesting the predictive benefit of ctDNA during the treatment course. In ongoing clinical trials across various tumor types, including CRC, the dynamics of ctDNA, as measured by changes in VAF percentage and/or ctDNA clearance, have emerged as important biomarkers.

The application of liquid biopsy-guided adjuvant therapy for CRC is still in the research stage and requires further clinical validation and optimization. However, it has the advantages of non-invasiveness, repeatability, and real-time monitoring, and is expected to become one of the important auxiliary tools for personalized treatment of CRC.

## 4 Opportunities and breakthroughs of liquid biopsy in the era of immunotherapy

Significant progress has been made in the study of CTCs and ctDNA using traditional liquid biopsy methods, which have played a powerful auxiliary role in tumor treatment (126–128). Immunotherapy has shown remarkable efficacy in various types of cancer, but it may impact the results of liquid biopsy. Therefore, it is necessary to reassess the traditional liquid biopsy criteria to accommodate the needs of immunotherapy (129, 130). Emerging detection technologies have provided support for liquid biopsy in optimizing treatment strategies, thus contributing to further advancements in the field of immunotherapy.

### 4.1 T Cell sorting: liquid detection based on flow cytometry

T-cell subset isolation is a method used to separate and purify T cells from a mixed population of cells. Flow cytometry can analyze various indicators such as T-cell subgroups, functional status, and expression of immune checkpoint molecules in blood samples. It can aid in evaluating a patient’s immune status and predicting the response to immune therapy (131–135).

The peripheral blood TCR repertoire serves as an important biomarker for the selection of ICIs therapy (136). TCR sequencing enables the study of the immune response mechanism of T cells. Longitudinal monitoring of the dynamic therapeutic evaluation of the TCR repertoire in ctDNA in peripheral blood provides insights into the co-evolution of tumors and immune components during ICIs treatment. TCR repertoire diversity, early conversion of peripheral T cells, and overall remodeling of the T-cell repertoire are associated with clonal regulation during ICIs treatment and are linked to anti-tumor immune responses. The presence of persistently exhausted TCR clones in peripheral blood is associated with adverse reactions to immune therapy (137–142). By combining flow cytometry and gene sequencing techniques, TCR sequences can be rapidly and accurately detected to understand T-cell clonal expansion and diversity.

Peripheral blood immune cell biomarkers, as one of the easily accessible biomarkers, can assess treatment response in the early stages and facilitate adjustments in early management (143–145). Studies have shown that the quantity and function of Tregs cells change in patients receiving immune therapy and are associated with poor prognosis (146–150). Studies using flow cytometry and RNA analysis have found that the percentage of circulating CD4+ and CD8+ T cells is associated with inflammatory tumors, indicating the significant role of these biomarkers in anti-tumor responses (151). Additionally, circulating T-cell lymphocyte subpopulations have been identified as biomarkers for mCRC (152). Decreased proportions of CD4+ cells and Tregs during treatment with folinic acid, 5-fluorouracil, and irinotecan (FOLFIRI) plus bevacizumab are associated with improved survival rates (153). Systemic immune inflammation index, ratios of different immune cells, and ratios of immune cells to platelets are also biomarkers for prognosis and prediction in CRC patients, including platelet-to-lymphocyte ratio (PLR) and neutrophil-to-lymphocyte ratio (NLR) (154–156). Recent studies have discovered novel circulating non-tumor cells and their biomarkers and extracellular matrix components, which have clinical application value in diagnosis, prognosis, and treatment response (157). Some studies suggest that circulating tumor endothelial cells (CTECs) from the tumor may play a prognostic role in CRC, with higher predictive value than CTCs (158–160). Similarly, in patients with mCRC receiving treatment with bevacizumab and chemotherapy, CECs and CD276-positive CTECs based on flow cytometry significantly increase (161). Studies have also shown that CXCR4-positive CECs are associated with longer PFS and OS, providing predictive value for mCRC patients receiving bevacizumab treatment (162–164).

Furthermore, single-cell sequencing technology (scRNA-seq) allows the study of gene expression and genetic variations at the individual cell level (165). The first immunotranscriptomic study based on scRNA-seq was conducted on CD4+ T cells infiltrating CRC. In this study, the impact of the tumor immune microenvironment (TIME) on specific gene expression (LAYN, MAGEH1, and CCR8) in tumor-infiltrating Tregs cells was confirmed, and these gene expressions were found to be correlated with immune therapy response, tumor-suppressive activity, and prognosis (166).

The gene characteristics of peripheral blood immune cells have received attention in the field of immune therapy and precision medicine. By combining T-cell subset isolation and liquid biopsy, comprehensive monitoring tools for immune therapy can be obtained, leading to a better understanding of tumor immune response and treatment outcomes, as well as optimization of treatment strategies.

### 4.2 Mass spectrometry techniques: unveiling the immunotherapeutic potential of neoantigens and non-mutated neoantigens

**Figure 4:**
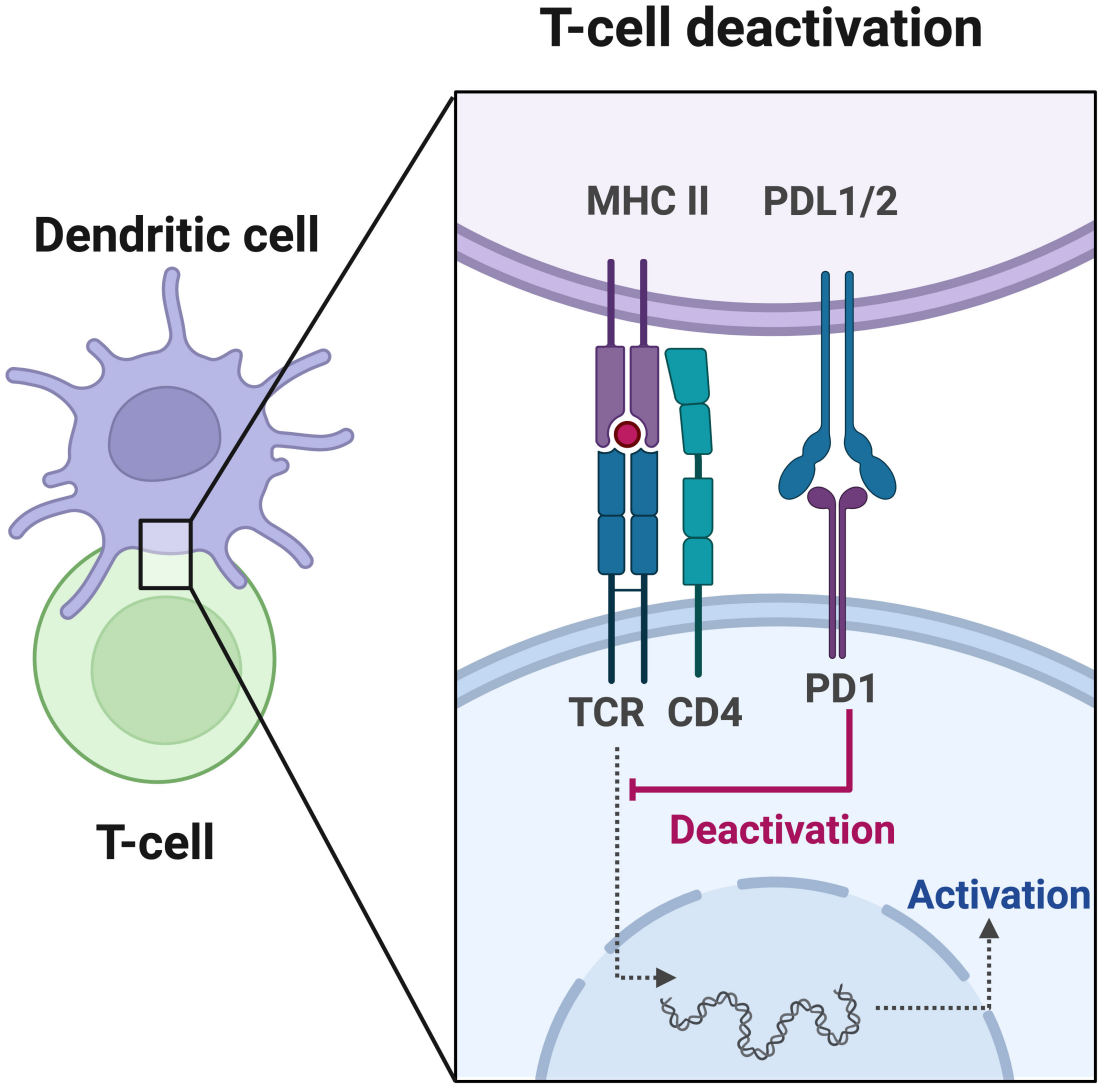
Neoantigens, which are mutated peptides generated through proteasome-mediated endogenous protein degradation, can subsequently be transported to the endoplasmic reticulum (ER) via antigen processing-associated transporter (TAP) and may be loaded onto MHC-I. In the ER, MHC-II dimers assemble and associate with the invariant chain (Ii). The Ii-MHC-II complex can be transported directly from the cell surface or, in some cases, indirectly endocytosed into the MHC-II compartment (MIIC). Within the MIIC, a series of endolysosomal proteases degrade Ii, releasing it and enabling MHC-II to bind specific peptide segments derived from mutated proteins within the endocytic pathway. These pMHC complexes are subsequently transported to the cell surface, where they are recognized by T cells.

Mass spectrometry-based liquid-phase detection is a novel technique that allows for the molecular-level monitoring of chemical components within cells and organisms, providing deeper insights into biological information. Neoantigens are novel antigenic epitopes generated by genetic mutations and serve as important targets in personalized immunotherapy (129). Neoantigens can be produced through proteasome-mediated endogenous protein degradation, and the resulting mutated peptides are subsequently transported to the endoplasmic reticulum (ER) via antigen processing-associated transporter (TAP), where they may be loaded onto MHC-I. MHC-II dimers assemble in the ER and associate with the invariant chain (Ii). The Ii-MHC-II complex can be transported directly from the cell surface or, at times, indirectly endocytosed into the MHC-II compartment (MIIC). Within the MIIC, a series of endolysosomal proteases degrade Ii, releasing it and enabling MHC-II to bind specific peptide segments derived from mutated proteins within the endocytic pathway. These peptide-MHC (pMHC) complexes are subsequently transported to the cell surface, where they are recognized by T cells (167) (**Figure 4**). Neoantigens possess potential high specificity and targeting, but they are predominantly patient-specific, making it challenging to categorize their utility, and they are often prominent in cancer patient populations. Currently, immune therapies, ICIs, tumor-specific vaccines, and neoantigen-based tumor-infiltrating lymphocytes (TILs) play increasingly important roles in cancer treatment (168). Studies have observed that certain CRC patients with MSI-H may benefit from ICIs treatment due to the presence of neoantigens (169). One of the main obstacles faced in personalized neoantigen immunotherapy is the accessibility of tumor biopsies. Thus far, the identification of neoantigens has typically involved genomic analysis of various tumor biopsies (170). Although this approach is time-consuming, invasive, and has a low positivity rate, it is more common in challenging cases requiring repeated sampling or when samples are limited, particularly in cases of frequent occurrence and metastatic cancers. Specifically, the presence of natural neoantigens at the top of immune checkpoints can enhance the effectiveness of significant inhibitors (171, 172). Given the current situation, liquid biopsies can serve as a viable alternative approach to identify potential neoantigens as immune therapeutic targets, applicable to numerous cancers. Although the limitations of detecting genomic mutations in plasma samples lie in detecting low allele frequencies, the reliability of genetic information obtained from liquid biopsies has been demonstrated (173). Therefore, based on current research on liquid biopsies, valuable insights can be provided for the use of neoantigens in treatment selection. Mass spectrometry-based liquid-phase detection allows for efficient identification and quantification of protein compositions within tumor cells, enabling the discovery of novel tumor-specific antigens by monitoring neoantigens in serum (174). Neoantigen-based immunotherapeutic approaches, such as ICIs, tumor-specific vaccines, and TILs, have become increasingly important in cancer treatment (168). Not all MSI-H CRC patients benefit from ICIs treatment; however, certain MSI-H colorectal cancer patients may benefit from ICIs treatment due to the presence of highly immunogenic neoantigens (169, 173).

Non-mutated neoantigens (NM-neoAgs) are immunogenic protein fragments generated through translational modifications or protein degradation of apoptotic tumor cells (175–181). These unique fragments do not exist in normal cells and are more easily processed and cross-presented by antigen-presenting cells (182). Studies utilizing mass spectrometry techniques and memory T cells as probes have identified NM-neoAgs in serum and found a strong correlation between high levels of NM-neoAgs and the efficacy of immunotherapy. Following induction chemotherapy, the response of NM-neoAgs-specific effector T cells (CD4+ and CD8+ T cells) increases and is further enhanced after immunotherapy, closely associated with patients’ survival rates and decreased expression levels of PD-1 (182). NM-neoAgs can target tumors with lower mutational burdens, contributing to the development of effective T cell-based immunotherapies for various cancer patients (182), and expanding the potential targets of liquid biopsy.

In summary, neoantigens and NM-neoAgs are tumor cell-specific antigens with tremendous potential in personalized immunotherapy. New detection methods such as flow cytometry and mass spectrometry techniques provide powerful tools for evaluating the efficacy of immunotherapy, thereby offering more effective treatment strategies for patients.

## 5 Conclusion

Liquid biopsy, as a non-invasive detection method, has emerged as a promising approach for early screening, diagnosis, postoperative monitoring, treatment response assessment, and evaluation of tumor resistance (183). With advancements in mass spectrometry-based detection of neoantigens and T cell sorting techniques such as flow cytometry, liquid biopsy has gained support as an adjunctive tool in the field of immunotherapy, providing opportunities for optimizing treatment strategies. However, despite significant progress, liquid biopsy remains in the exploratory and developmental stage, facing various challenges and complexities.

These include issues including the typically low concentrations of analytes collected from samples (184, 185), lack of standardization and uniformity for liquid biopsy biomarkers, and a dearth of widely accepted clinical practice guidelines (186, 187),related to false-positive results (188), variations in sensitivity among studies (82, 189), limitations in detection sensitivity and specificity (186, 190), and susceptibility to interference (184, 189, 191, 192). Overcoming these challenges and advancing liquid biopsy requires the development of highly sensitive and specific detection methods, standardization of experimental procedures and validation methods, and the application of artificial intelligence and machine learning algorithms for data analysis and interpretation. Additionally, the exploration of new biomarkers and the conduct of large-scale multicenter studies and clinical trials are essential to enhance the accuracy of early diagnosis and treatment prediction (193–196).

Despite the challenges that remain, the potential of liquid biopsy-assisted immunotherapy in transforming the field of immunotherapy is undeniable. Looking ahead, in the era of immunotherapy, liquid biopsy-assisted immunotherapy has the potential to fundamentally change the field and provide patients with more precise, effective, and personalized treatment strategies. Continued research, clinical trials, and technological advancements will play a crucial role in fully harnessing liquid biopsy as a valuable tool for guiding immunotherapy and improving future patient outcomes.

## Author contributions

SY: Conceptualization, Data curation, Formal analysis, Writing – original draft. YH: Investigation, Methodology, Writing – review & editing. MY: Resources, Software, Writing – original draft. KJ: Conceptualization, Data curation, Writing – original draft, Writing – review & editing. HL: Investigation, Software, Validation, Writing – review & editing.

## Glossary

**Table:**

CRC	Colorectal cancer
ICIs	immune checkpoint inhibitors
CTCs	circulating tumor cells
ctDNA	circulating tumor DNA
cfDNA	circulating cell-free DNA
MRD	minimal residual disease
mCRC	metastatic colorectal cancer
DFS	disease-free survival
PFS	progression-free survival
OS	overall survival
LARC	locally advanced rectal cancer
MSI-H	MSI-H microsatellite instability
CAR-T	chimeric antigen receptor T
TCR	T cell receptor
PLR	platelet-to-lymphocyte ratio
NLR	neutrophil-to-lymphocyte ratio
CTECs	circulating tumor endothelial cells
scRNA-seq	single-cell sequencing technology
TILs	tumor-infiltrating lymphocytes
TIME	tumor immune microenvironment
NM-neoAgs	Non-mutated neoantigens
neoAgs	Neoantigens
FDA	Food and Drug Administration
MSI-H	microsatellite-high
dMMR	deficient mismatch repair
PD-L1	programmed cell death ligand 1
PD-1	programmed death receptor 1
RFS	recurrence-free survival
NGS	next-generation sequencing
RFS	recurrence-free survival
IHC	immunohistochemistry
PCR	polymerase chain reaction
ORR	overall response rates
TME	tumor microenvironment
TMB	tumor mutational burden
MSS	microsatellite-stable
IHC	immunohistochemistry
PCR	polymerase chain reaction
pMMR	proficient mismatch repair
ER	endoplasmic reticulum
TAP	processing-associated transporter
Ii	invariant chain
MIIC	MHC-II compartment
pMHC	peptide-MHC
